# Prevalence of *Escherichia coli* in Under-Five Children with Diarrhea in Ethiopia: A Systematic Review and Meta-Analysis

**DOI:** 10.1155/2020/8844294

**Published:** 2020-09-07

**Authors:** Tizazu Zenebe, Meseret Mitiku, Yonas Alem

**Affiliations:** ^1^Department of Medicine, Medical Microbiology Unit, DebreBerhan University, DebreBerhan, Ethiopia; ^2^Department of Medical Laboratory Sciences, MaddaWalabu University, Bale Robe, Ethiopia; ^3^Department of Medical Laboratory Sciences, Ambo University, Ambo, Ethiopia

## Abstract

Diarrhea remains as a high health burden, especially to children in low-income countries including Ethiopia. Diarrheagenic *Escherichia coli* have been commonly associated as bacterial pathogens causing diarrheal disease among children. This systemic review and meta-analysis was intended to determine the pooled prevalence of *Escherichia coli* in under-five children with diarrhea in Ethiopia. A comprehensive search in PubMed, Google Scholar, ScienceDirect, ResearchGate, and Google search engine and manual searching were done for this systematic review and meta-analysis. The eligibility criteria for selecting studies were studies involving under-five children with diarrhea in Ethiopia, published articles, cross-sectional studies, and articles reported in English. The study was conducted based on the Preferred Reporting Items for Systematic Reviews and Meta-Analysis (PRISMA) checklist. The data analysis was done using STATA 16.0 software. Cochran's *Q*-test and *I*^2^ statistics were used for the assessment of heterogeneity. The random-effect model was used to estimate the pooled prevalence of *Escherichia coli*. A total of 797 articles were initially retrieved, and finally, 11 studies met the eligibility criteria and were included in the final meta-analysis. The pooled prevalence of *Escherichia coli* was 25% (95% CI: 9, 41). The pooled prevalence was varied by region, detection method, and sample size. The high prevalence emphasizes that *Escherichia coli* is a potential pathogen in under-five children with diarrhea in Ethiopia.

## 1. Introduction

The World Health Organization (WHO) defined diarrhea as the passage of three or more loose or liquid stools per day [1]. Due to the complex interplay of the environment, food, water, and sanitation with poverty and deprivation, diarrhea remains as a high health burden, especially to children in the world [2]. Diarrhea was one of the top ten causes of death, ninth globally, sixth in lower-middle-income countries, and the second in low-income countries in 2016 [3]. Ethiopia ranks sixth with the highest burden of pneumonia and diarrhea in the world [4]. National data [5], many local studies [6–10], and other reports [11] showed that diarrhea is still a common health problem in the country.

Diarrhea could be caused by different enteric pathogens which include bacteria, viruses, and parasites [12]. Diarrheagenic *Escherichia coli*, *Campylobacter, Shigella, Vibrio cholerae, Salmonella*, and *Clostridium difficile* are the common bacterial agents of diarrhea [12, 13]. Diarrheagenic *E. coli* have been predominantly associated as bacterial pathogens causing diarrheal disease among children in developing countries [14]. There are many local studies done on enteropathogens that showed bacterial pathogens causing diarrhea in under-five children in Ethiopia [15–17]. There are also studies done on *E. coli* in under-five children in the country [18–23]. However, there are no data on the pooled prevalence of *E. coli* at the national level in under-five children in Ethiopia. Therefore, this systemic review and meta-analysis was intended to determine the pooled prevalence of *E. coli* in under-five children in Ethiopia. And the comprehensive evidence will not only have an impact on control and prevention but also indicates the importance of the pathogens and the need of further studies in Ethiopia.

## 2. Methods

### 2.1. Study Design

A systematic review and meta-analysis was conducted to estimate the pooled prevalence of *E. coli* in under-five children with diarrhea in Ethiopia. The study followed a similar approach with Alebe et al. [24] and was conducted based on the PRISMA checklist [25].

### 2.2. Literature Search Strategies for Relevant Studies

A comprehensive search with no date limits was performed in the following databases: PubMed, Google Scholar, ScienceDirect, ResearchGate, and Google search engine, and by manual searching. The search of the literature was conducted between March 1 and April 30, 2020. All articles published until April 30, 2020, were considered for conducting the review. *E. coli*, Under-five Children, Diarrhea, diarrhoea, and Ethiopia were used as search arm. We use search terms using Boolean operators for PubMed as follows: (((“*Escherichia coli*”) *AND* (*Children*)) *AND* (“*Diarrhea*” *OR* “*diarrhoea*”)) AND (*Ethiopia*) were used for searching. We also use Boolean operators for Google Scholar and ScienceDirect as follows: “*Escherichia coli*” and “Under-five” and “Children” and “diarrhea” or “diarrhoea” and “Ethiopia.”

### 2.3. Study Eligibility Criteria

All available studies and data were incorporated based on the following predefined eligibility criteria. The inclusion criteria were studies conducted in under-five children with diarrhea in Ethiopia, published articles, cross-sectional study, and articles reported in English. The exclusion criteria were articles with duplicate or overlapping data and without full text available.

### 2.4. Study Selection

Records identified from various sources with the search strategies were exported to Endnote reference software version 7. Duplicate records were identified, recorded, and removed with Endnote and manually. For this, two authors independently screened the title and abstracts with the predefined inclusion criteria. Two authors also independently collected full texts and evaluated the eligibility of studies for final inclusion. When discrepancies between two authors occur, the other reviewers solve it through discussion and consensus.

### 2.5. Measurement of Outcome Variables

The outcome of the study was the pooled prevalence of *E. coli* in under-five children with diarrhea. The prevalence was calculated by dividing the numbers of bacterial isolates by the total number of clinically related patients. In other words, *E. coli* isolation rate was defined as the ratio of the number of positive samples for bacteria to the total number of sample size (negative and positive for bacteria).

### 2.6. Data Extraction

Data from selected articles were extracted using a standardized data extraction format, by two authors independently extracting all necessary data. Any disagreement during the data extraction was resolved through discussion and consensus. The primary author of the original research was contacted for additional information or to clarify method details as needed. The data extraction format included primary author, publication year, region, study area, sample size, detection method, and prevalence of *E. coli*.

### 2.7. Quality Assessment

Two authors independently assessed the risk of bias for each original study. The quality of the study was evaluated according to the Newcastle–Ottawa scale adapted for cross-sectional studies [26] and graded out of 10 points (10 stars). The tool has three major sections: methodological quality (with 5 stars), comparability of the study (with 2 stars), and outcomes related to statistical analysis (with 3 stars). The mean score of two authors with a third author negotiation was taken for final decision, and studies with a score greater than or equal to five were included.

### 2.8. Data Processing and Analysis

The relevant data were extracted from selected studies using format prepared in Microsoft Excel. The data analysis was done using STATA 16.0 software. The data entered into Microsoft excel were imported to the STATA for outcome measures and subgroup analyses. For the determination of variation in true effect sizes across population (clinical heterogeneity), a restricted maximum-likelihood random-effect model was applied. The original articles were described using forest plot, funnel plot, and tables. The random-effect model was used to compute the pooled prevalence of *Escherichia coli.* The estimated pooled prevalence with 95% confidence intervals by forest plot and publication bias by funnel plot were presented.

Subgroup analysis was performed based on region, publication year, detection method, and sample size. Studies from Addis Ababa City Administration, Tigray, and Amhara regional state were included, and a study from other regions was not available. The publication year was categorized into three: 1970–1990 (two decades), 1991–2010 (two decades), and 2011–2020 (a decade). The detection methods were grouped as follows: culture only, serotyping, serotyping and enterotoxin test, and molecular method.

Heterogeneity among reported prevalence was assessed by computing *p* values of Cochran's *Q*-test and *I*^2^ statistics [27]. Cochran's *Q*-test evaluates the existence of heterogeneity, and *p* < 0.10 indicates statistically significant heterogeneity [28]. The *I*^2^ statistics provides an estimate of the percentage of the variability in effect estimates that is due to heterogeneity rather than sampling error or chance differences. *I*^2^ values of 25%, 50%, and 75% are considered to represent low, medium, and high heterogeneity, respectively [27, 28]. Begg's rank test and Egger's regression test are among various statistical tests used for checking publication bias in the funnel plot [29]. Begg's rank test examines the correlation between the effect sizes and their corresponding sampling variances. And a strong correlation implies publication bias. Egger's test regresses the standardized effect sizes on their precisions; in the absence of publication bias, the regression intercept is expected to be zero [29]. Egger's test at 5% significant level was done to check publication bias [29, 30].

## 3. Results

**Figure 1:**
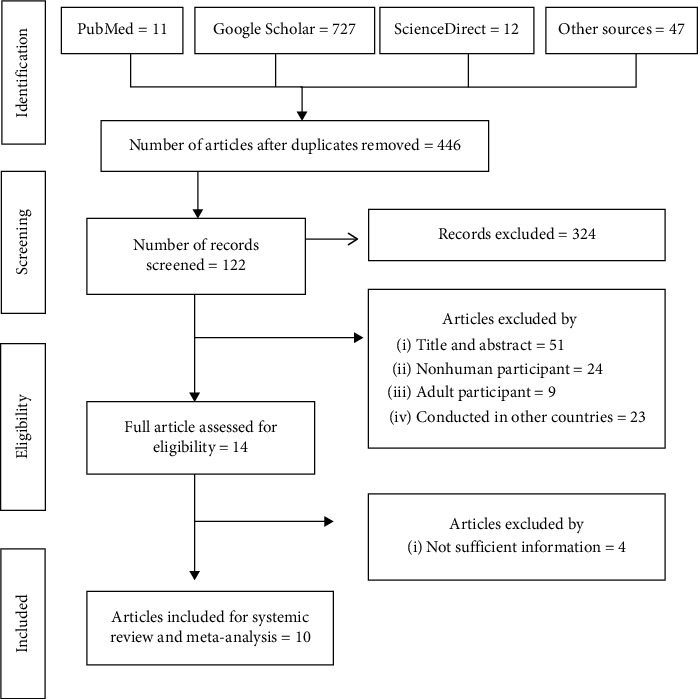
Flowchart of study selection for systematic review and meta-analysis of the prevalence of *Escherichia coli* in under-five children with diarrhea in Ethiopia.

A total of 797 articles were initially retrieved for the prevalence of *E. coli* in under-five children with diarrhea using the range of databases described above. Among the total, 351 articles were removed after duplicates, and again after screening, 324 articles were excluded. Moreover, 108 articles were excluded based on title and abstract, involving nonhuman and adult participants, inaccessibility of full texts, and studies conducted in other countries. And also, 4 more articles were excluded due to insufficient information. Finally, 10 studies met the eligibility criteria and were included in the final meta-analysis (Figure 1).

### 3.1. Description of Search Results

All the included 10 articles (Table 1) used a cross-sectional study design and were published from 1976 to 2019, data in the last 4 and more decades. A total of 2269 (ranging from 98 to 422) study participants were involved to determine the pooled prevalence of *E. coli* among under-five children with diarrhea. These studies were conducted in Addis Ababa City Administration and Amhara regional state, Ethiopia. Majority of the studies (8 studies) were done in Addis Ababa [19, 21–23, 31–33], and the remaining 2 were done in Amhara regional state [17, 18]. Three of the studies [21, 22, 31] were very old (published before 2000) compared to the others. The lowest prevalence was 20.8% [19] and the highest prevalence was 62.7% [17]. There were no reported data from the other regional states and city administration in Ethiopia during the preparation of the present review.

### 3.2. Prevalence of *Escherichia coli* in Under-Five Children

**Figure 2:**
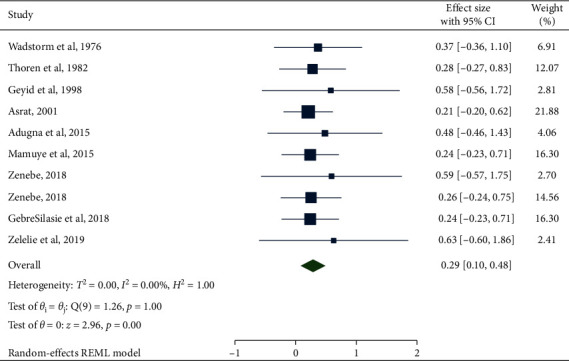
Forest plot of the pooled prevalence of *Escherichia coli* in under-five children with diarrhea in Ethiopia.

**Figure 3:**
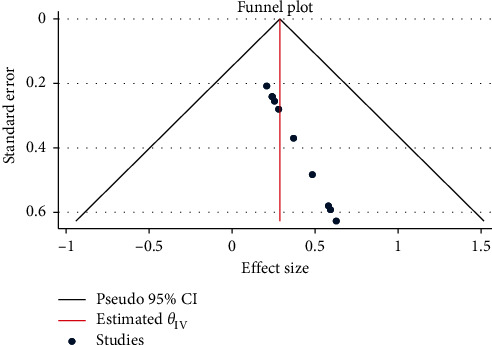
Funnel plot with 95% confidence limits of the pooled prevalence of *Escherichia coli* in under-five children with diarrhea in Ethiopia.

In the present systemic and meta-analysis, the pooled prevalence of *E. coli* was 29% (95% CI: 10, 48). There was no heterogeneity observed across the included studies (*I*^2^ = 0.00%; *Q* = 1.84, *p*=1.00). Regardless of the absence of heterogeneity, the random-effect model was used to estimate the pooled prevalence of *E. coli* in under-five children with diarrhea in Ethiopia (Figure 2). In this meta-analysis, the lowest prevalence was 20.8% [19] and the highest prevalence was 62.7% [17]. To check for the presence of publication bias, funnel plot asymmetry was used (Figure 3). The funnel plot showed asymmetry, which was indicative of the presence of publication bias. However, Egger's tests showed that there was no statistically significant publication bias in estimating the prevalence of *E. coli* in under-five children (*p*=0.2611).

### 3.3. Subgroup Analysis

A subgroup analysis for region, publication year, method of detection, and sample size was done. A higher estimate of the pooled prevalence of *E. coli* (54, 95% CI: -0.21, 1.29) was observed in Amhara regional state (Table 2). Almost similar estimate of the pooled prevalence of *E. coli* (21%) was observed across the two last decades. A higher prevalence of *E. coli* was detected through serotyping and enterotoxin test (31% with 95% CI: −12, 75). Studies with sample size greater than 200 have higher estimated pooled prevalence (26% (95% CI: 2, 49)) compared to those with sample size less than or equal to 200.

## 4. Discussions

*E. coli* are not only important member of the normal intestinal microbiota of humans and other mammals but also they are highly specialized pathogenic strains causing worldwide outbreaks of severe diseases and opportunistic pathogens [34, 35]. Mainly there are six intestinal pathotypes [34, 36]. These are enteropathogenic *E. coli*, Shiga toxin-producing *E. coli*, enterotoxigenic *E. coli*, enteroinvasive *E. coli*, enteroaggregative *E. coli*, and diffusely adherent *E. coli*. However, identifying such pathogenic or diarrheagenic *E. coli* strains needs molecular techniques such as polymerase chain reaction (PCR) [36]. In the present systemic and meta-analysis, majority of the included studies used conventional methods for the detection of *E. coli*. This means that the reported prevalence of *E. coli* in these studies may not be necessarily true pathogen or diarrheagenic *E. coli.* A bacteriological analysis shows that 100 water samples (50 from cases and 50 from controls) were found to be positive for total coliforms [37]. In the study, 70% of sampled water from cases and 74% of sampled water from controls were positive for *E. coli* [37]. This supports that *E. coli* may not be necessarily the cause of the disease case (diarrhea) unless confirmed by advanced laboratory techniques.

The estimated pooled prevalence of *E. coli* was 25% in the present study which could be considered as a higher prevalence rate. Except the two studies [23, 31], all were done with conventional detection methods that could contribute to the higher prevalence of the bacteria (nonpathogenic versus pathogenic). The present pooled prevalence of *E. coli* was higher compared to the pooled prevalence of *Shigella* species (6.6%) reported in systemic review and meta-analysis done in Ethiopia [38]. In a systemic and meta-analysis study done in foods of animal origin (beef, milk, mutton, chevon, and chicken) and environmental swabs in Ethiopia, the estimated pooled prevalence of *E. coli* was 15% [39] which is less than that reported in the present study. On the contrary, meta-analysis study done on *E. coli* O157 : H7 found 4% prevalence in foods of animal origin [40] and 5% pooled prevalence of *E. coli* O157 : H7 [41]. This discrepancy may be due to difference in study subjects, and detection method, or true prevalence.

In the subgroup analysis, the estimated pooled prevalence of *E. coli* was varied by region, sample size, and detection method. All studies were done in Addis Ababa City Administration and Amhara regional state. The estimated pooled prevalence is relatively higher in Amhara regional state (54%) than in Addis Ababa (27%). It may be due to difference in access to improved source of drinking water, 97% access to urban households and 57% to rural households [5]. And also difference in essential hand-washing agents, the availability of soap and water, is highest in Addis Ababa (39%) and lowest in Amhara (5%) [5]. In addition, difference in diagnosis method used, actual prevalence difference, geographical location, and difference in the number of studies involved in the analysis could make the difference. Still the prevalence in Addis Ababa is higher (27%). This may be due to the sanitation problem according to a report by Adane et al. [6] which indicated that 94.6% of unimproved sanitation facilities and greater intermittent water supply [42] occurred in Addis Ababa. In both study areas, the higher prevalence signifies the need for molecular characterization of pathogenic strains for a better understanding of prevention and control strategies.

The prevalence of *E. coli* is almost unchanged through time (over 43 years). It may be indicative of the absence or lack of interventions against the infection of *E. coli* or it may be neglected among common communicable disease-causing agents. In terms of detection methods, the higher prevalence was seen by serotyping and enterotoxin test (31%). In contrary to the reality, the lower prevalence was reported using the culture detection method (19%). According to a report by Zenebe [23], the prevalence of *E. coli* was 59.2% using the culture method, but with PCR it was 25.5% which is expected. Thus, the inconsistency might be due to the number of studies included in the analysis for each method. The estimated pooled prevalence of *E. coli* was 35% for sample size equal to or less than 200 which is relatively higher than for sample size greater than 200 (26%) but not statically significant.

The main limitation of the present study is that majority of the studies were done using the culture detection method only which could not rule out pathogenic *E. coli* from nonpathogenic. In addition, few numbers of articles were included mainly due to less number of studies done in Ethiopia. And also, the absence of data from other regional states and city administration could be considered as another potential limitation of the study.

## 5. Conclusions

Regardless of differentiating and assuring the pathogenic from nonpathogenic, a high prevalence of *E. coli* was observed in under-five children with diarrhea in Ethiopia. The study highlighted that *E. coli* may be considered as a potential pathogen in under-five children with diarrhea in Ethiopia. The finding justifies the need for molecular characterization and further epidemiological studies which might be important for searching preventive and intervention strategies. And also, generating data from other regional states and city administration could be useful for a full-blown picture of the problem at a country level.

## Figures and Tables

**Table 1:** Summary of selected studies reporting the prevalence of *Escherichia coli* in different parts of Ethiopia, from 1970 to 2019.

Study	Quality score	Publication year	Region	Study area	Sample size	Detection method	Prevalence of *E. coli* (%)
Wadstrom et al. [22]	7	1976	Addis Ababa	Addis Ababa	354	Serotyping and enterotoxin test	37
Thoren et al. [21]	6	1982		Addis Ababa	175	Serotyping and enterotoxin test	28
Geyid et al. [31]	5	1998		Addis Ababa	108	DNA probe	58
Asrat [19]	5	2001		Addis Ababa	345	Serotyping	20.8
Mamuye et al. [32]	5	2015		Addis Ababa	253	Culture	24.1
Zenebe [23]	5	2018		Addis Ababa	98	Culture	59.2
Zenebe [23]	5	2018		Addis Ababa	98	Culture	25.5
GebreSilasie et al. [33]	5	2018		Addis Ababa	253	Culture	24.1

Zelelie et al. [17]	5	2019	Amhara	Debre Berhan	163	Culture	62.7
Adugna et al. [18]	6	2015		Bahr Dar	422	Serotyping	48.3

**Table 2:** Subgroup estimate of the pooled prevalence of *Escherichia coli* in under-five children with diarrhea in Ethiopia.

Variables	Characteristics	Included studies	Sample size	Prevalence of *E. coli* (95% CI)
Region	Addis Ababa	8	1684	27 (7, 47)
	Amhara	2	585	54 (−0.21, 1.29)

Publication year	1976–1990	3	637	35 (−6, 76)
	1991–2010	1	345	21 (−20, 62)
	2011–2019	6	1356	21 (3, 39)

Detection method	Serotyping	2	767	25 (−12, 63)
	Serotyping and enterotoxin test	2	529	31 (−12, 75)
	Culture only	5	934	19 (0, 38)
	Molecular method	2	206	31 (−15, 77)

By sample size	≤200	6	809	21 (1, 40)
	>200	5	1627	26 (2, 49)

## Data Availability

The datasets used and/or analyzed during the current study are available from the corresponding author on reasonable request.
